# Impact of COVID on the medical activity of occupational health departments

**DOI:** 10.1371/journal.pone.0323018

**Published:** 2025-05-22

**Authors:** Luther Dogbla, Amine Ben Jaber, Julien S. Baker, Gil Boudet, Ilhem Karoui, Ahmed Hajji, Asma Korbi, Ukadike Chris Ugbolue, François-Xavier Lesage, Marek Zak, Aurélien Mulliez, Frédéric Dutheil

**Affiliations:** 1 Université Clermont Auvergne, CNRS, LaPSCo, Physiological and Psychosocial Stress, University Hospital of Clermont–Ferrand, CHU Clermont-Ferrand, Occupational and Environmental Medicine, Clermont-Ferrand, France; 2 SSTI03, Service de Santé Interentreprise de l’Allier (Inter-Enterprise Occupational Health of Allier), Vichy, France; 3 Hong Kong Baptist University, Sport and Physical Education, Kowloon, Hong Kong; 4 Université Clermont Auvergne, CNRS, LaPSCo, Physiological and Psychosocial Stress, Clermont-Ferrand, France; 5 University Hospital of Monastir, Gynecology and Obstetrics, Monastir, Tunisia; 6 University of the West of Scotland, School of Health and Life Sciences, Institute for Clinical Exercise & Health Science, Glasgow, United Kingdom; 7 Institut Desbrest of Epidemiology and Public Health, INSERM and University of Montpellier, Montpellier, France Behaviors, CHU Montpellier, Occupational Medicine, Montpellier, France; 8 The Jan Kochanowski University, Faculty of Medicine and Health Sciences, Institute of Physiotherapy, Kielce, Poland; 9 University Hospital of Clermont Ferrand, CHU Clermont-Ferrand, Biostatistics, Clinical Research and Innovation Direction, Clermont-Ferrand, France; CHUV: Centre Hospitalier Universitaire Vaudois, SWITZERLAND

## Abstract

**Background:**

To determine the impact of the Covid-19 pandemic on the number of occupational health consultations and to highlight influencing factors.

**Method:**

Retrospective observational study of consultations from an inter-company occupational health service. Data were retrieved during three consecutive years: 2019 (baseline), and 2020–2021. For comparisons purposes, we used the number of occupational health consultations per day and per full-time equivalent occupational healthcare worker (n consultations/d/FTE). Multivariate analysis was performed using logistic regression, for each lockdown vs the same period one year before.

**Results:**

A total of 103,351 consultations were included. The number of consultations decreased by 14.3% in 2020 compared to 2019 but increased by 33.7% in 2021 compared to 2020. There were 4.9 consultations/d/FTE, 4.69 to 5.12 in 2019; 4.07, 3.81 to 4.34 in 2020; and 5.35, 5.16 to 5.55 in 2021. The first lockdown had a massive impact on the number of consultations, whereas the activity returned to normal from August 2020 with an increase in 2021. Age was associated with a decrease in the propension of consulting for the three lockdown periods (p < 0.001). The proportion of consultations for return-to-work was multiplied by 2.44 (2.02 to 2.95, p < 0.001) during the first lockdown, associated with a reduced risk of being declared unfit to work (OR = 0.48, 95 CI 0.27 to 0.84, p = 0.010).

**Conclusion:**

The Covid-19 pandemic had a huge impact on the medical activity of occupational health departments, with a massive decrease in 2020 followed by an increase in 2021 compared to 2019.

## Introduction

During the Covid-19 pandemic, activity in the health sector underwent a significant reshaping worldwide [1]. A decrease in activity was found in several departments such as emergency departments [2–4], urology [5], ENT [6], ophthalmology [7], psychiatry [8], cardiology, or preventive health [9]. In occupational health, a Polish study revealed a 22% drop in the number of consultations compared to the year before the pandemic [10]. Despite a large number of consultations [11,12], no studies reported the influence of the Covid-19 pandemic on medical activity of occupational health departments. Occupational health, a crucial facet of healthcare, encompasses the specialized domain dedicated to safeguarding and enhancing the well-being of employees within their work environments [13]. If some research identified high users of primary care [14], such data are not yet available for patients consulting in occupational health medicine. Variables such as age and sex might be important factors influencing the demand for consultations in occupational health medicine [15]. Vulnerability or diseases [16–18] and occupational activities such as essentials sectors [19], have been shown to be strong determinant of the severity of the Covid-19 infection, and thus may also influence the demand for occupational health consultations during the first lockdown of the pandemic.

Therefore, we aimed to study the evolution of the number of consultations over a 3-year period (the year 2019 preceding the Covid-19 pandemic as reference year and the two years 2020–2021 of the pandemic). Secondary objectives were to assess the factors influencing consultation such as sociodemographic of workers (age, sex, vulnerability), characteristics of consultation (nurses, essentials sectors, return to work), and conclusions of consultations (consultation with orientation, workstation layout, unfit to work).

## Method

### Study design

This retrospective study was carried out in the inter-company occupational health service of Allier (Service de Santé Inter-entreprises de l’Allier, SSTI03). The SSTI03 in Allier operates as part of France’s mandatory occupational health framework, ensuring health consultations for employees across companies of all sizes. Subsidized by employers and the national health system, this service caters to diverse enterprises, and is accessible irrespective of company scale. The SSTI03 monitors an average of 80,000 employees per year. Date from consultations are collected into the PREVENTIEL software. We included all consultations conducted from three consecutive years, i.e., from the 1^st^ January 2019–31^st^ December 2021. The year 2019 is the control year, the year 2020 is the first year of the pandemic, and the year 2021 is the second year following the onset of the SARS-CoV-2. The dates of the 3 lockdowns in France were from 17 March to 10 May 2020 (first lockdown), from 30 October to 14 December 2020 (second lockdown), and from 3 April to 2 May 2021 (third lockdown). This study was approved by the appropriate ethics committee (Comité de Protection des Personnes Sud-Est VI, Clermont-Ferrand, France; No. 2015/CE 70).

### Outcomes

The primary outcome was the number of occupational health consultations per day and per full-time equivalent occupational healthcare worker (n consultations/d/FTE). Secondary outcomes were characteristics of employees, characteristics of consultations, and conclusions of consultations. Characteristics of employees who underwent consultations: age (continuous by decades), gender (male/female), and disabled worker status (benefiting or not from the employment obligation for disabled workers). The characteristics of the consultations were: consultations by physician or nurse, company sectors of activity (essential or non-essential) and type of consultations (return to work consultation or not). The company sectors are classified into the PREVENTIEL software using the statistical classification of economic activities in the European community 2008 (Nomenclature statitistiques des Activités économiques dans la Communauté Européenne – NACE 2008) level of aggregation 17 (A17), allowing to identify essential sectors of activity. We considered the following sectors are “essential”: health-care, mass distribution, and transport [19]. Conclusions of consultations were evaluated by: orientation after the consultation (none, or orientation by the occupational physician to the general practitioner or a specialist, orientation made by the nurse to the occupational physician, to the occupational psychologist, and to the partners helping for social insertion of workers), workstation layout (none, or adaptation prescribed by the occupational physician that cover equipment, supplies, accessories, or movements and postures), and unfit to work (none, or an unfit to work diagnosis prescribed by the occupational physician, i.e., the fact that the worker cannot work anymore at his workstation).

### Statistical analysis

Statistics were performed with Stata software (version 15, StataCorp, College Station, US). Data are described as frequency and percentage for categorical criteria and as means, standard deviation, median, and interquartile range for continuous criteria. Comparison between the 1^st^ lockdown period (17 March 2020–10 May 200) vs the same period one year before was performed using a chi-square test (or Fisher exact test when appropriate) for categorical variables and with Student’s t-test (or Mann-Whitney test if data were not normally distributed) for continuous variables, and Hedge’s bias corrected effect size was computed with its 95% confidence interval (95 CI). Effect-sizes are considered small between 0.20 and 0.50, moderate between 0.50 and 0.80, and high above 0.80 [20]. Multivariate analysis was performed using logistic regression. The period status (consultations during the 1st lockdown vs the same period one year before taken as control period) was considered as the dependent variable. Age of workers (continuous by decades), gender (sex ratio, i.e., male/female), workers with handicap (present/absent), consultation (physician/nurse), essential sector (true/false), return to work consultation (true/false), consultation with orientation (true/false), workstation layout (true/false) and unfit to work (true/false) were considered as independent factors in order to show the consultation’s characteristics related to changes between the control period and the 1^st^ lockdown period. Results are shown as odds ratio and their 95 CI. Those analyses were performed for all consultations, and into subgroups of medical consultation and nurse consultations. Same analyses were performed for the 2^nd^ lockdown period (vs same period one year before) and 3^rd^ lockdown (vs same period two years before). Evolution of consultation number (physician + nurse and total) were summed by day and plotted using 30 days moving average. All tests were 2-sided and a p-value <5% was considered statistically significant.

## Results

### Consultations included

**Fig 1:**
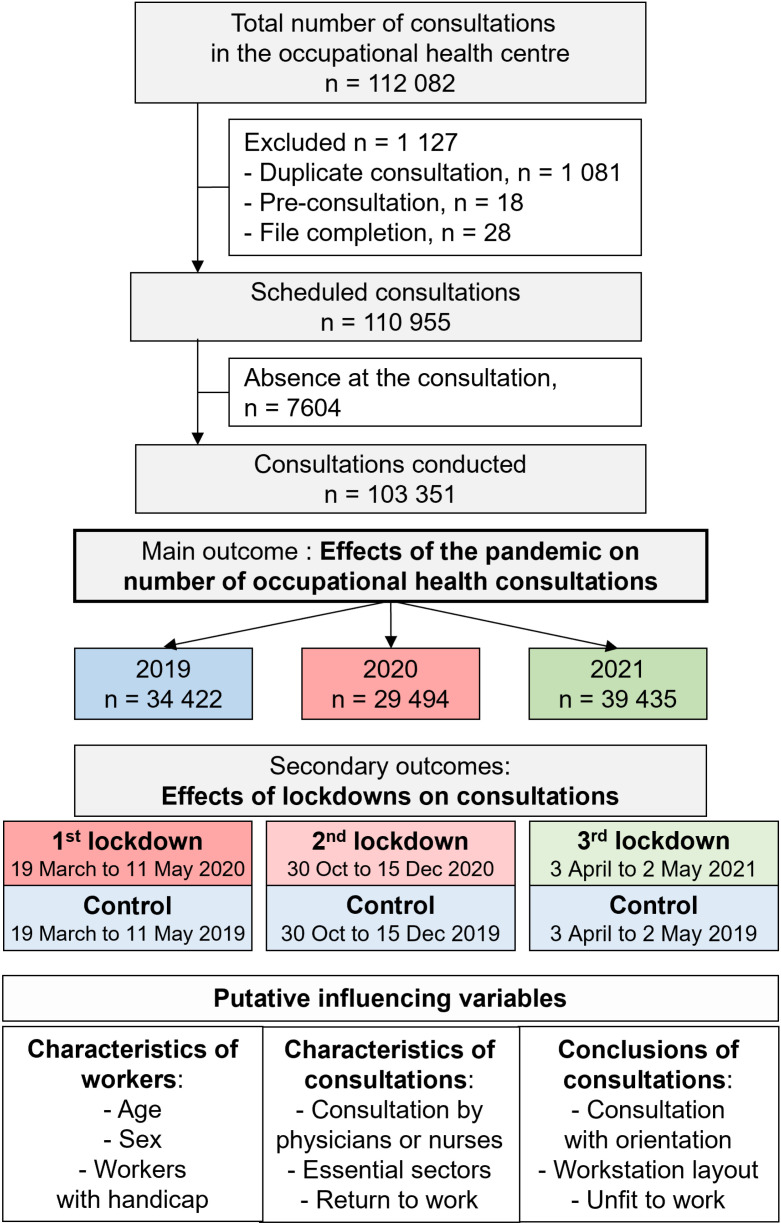
Flow chart.

From 1^st^ January 2019–31^st^ December 2021, the number of consultations extracted from the PREVENTIEL software was 112,082. After removal of duplicates and removal of pre-consultations (consultations conducted before the consultation to prepare the consultation), 110,955 were scheduled consultations. Due to absence at the consultation, 7604 consultations were excluded, resulting in a final sample of 103,351 consultations conducted and included for the main outcome (Fig 1). Workers who underwent those consultations were 41.1 ± 13.1 years old, 56.3% males, and 6.3% had a handicap. Regarding characteristics of consultations: 55.0% were done by physicians, 39.9% were in essential sectors, and 15.9% were return-to-work consultations. Regarding conclusions of consultations: 8.0% of workers had an orientation after the consultation (to another physician, a psychologist, or for social insertion), 8.0% had a workstation layout, and 3.5% had an unfit to work diagnosis.

### Evolution of the number of consultations over the years

**Fig 2:**
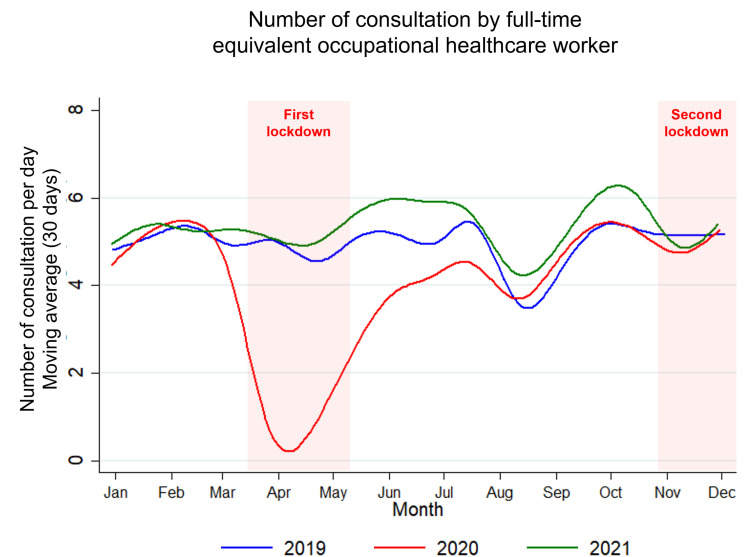
Number of consultations by full-time equivalent occupational healthcare worker.

Compared to 2019, the number of consultations decreased by 14.3% in 2020 (from 34,422–29,494), but increased by 14.6% in 2021 (from 34,422–39,435). In 2021, the number of consultations also increased by 33.7% compared to 2020 (39,435 vs. 29,494) (Fig 1). More specifically, the average of the number of consultations per day and per full-time equivalent occupational healthcare worker was the lowest in 2020 (4.07 consultations/d/FTE, 95 CI 3.81 to 4.34) – compared to 2019 (4.9 consultations/d/FTE, 95 CI 4.69 to 5.12) and 2021 (5.35 consultations/d/FTE, 95 CI 5.16 to 5.55), and was higher in 2021 compared to 2020 (p < 0.001, all absolute effect sizes being >0.80 for all comparisons) (S1 Table and Fig 2). More specifically, those conclusions are similar for nurses, whereas physicians had a decreased number of consultations mainly in 2020 (S1 Fig).

### Comparisons between lockdown periods

The average number of consultations per day dropped massively (effect size ‐3.32, 95 CI ‐4.02 to ‐2.61) during the 1^st^ lockdown (0.64 consultations/d/FTE, 95 CI 0.44 to 0.83) compared with the same period in 2019 (4.78, 95 CI 4.23 to 5.32). When comparing monthly data, the number of consultations returns to normal in August 2020 (compared to 2019). Then, there was also a slight decrease (effect size -0.03, 95 CI -0.52 to 0.47) during the 2nd lockdown (5.1 consultations/d/FTE, 95 CI 4.53 to 5.68) compared with the same period in 2019 (5.15, 4.6 to 5.69) (S1 Table and Fig 2). These same conclusions can be found for consultations made by physicians and by nurses (S1 Fig).

### Socio-demographic characteristics of employees

**Fig 3:**
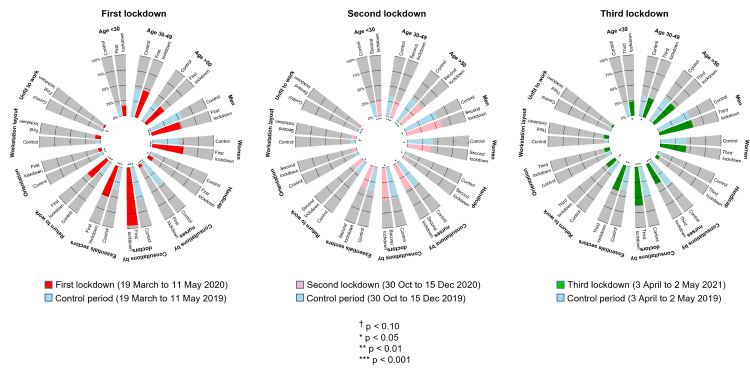
Prevalence of consultations (%) during each lockdown compared to a control period (one year before for the first and second lockdown, and two years before for the third lockdown), depending on characteristics of workers, characteristics of consultations, and conclusions of consultations.

**Fig 4:**
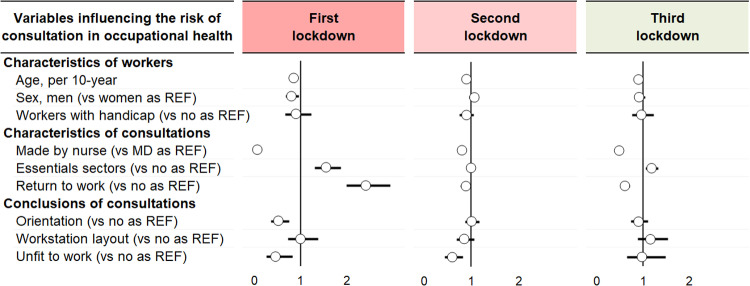
Variables influencing the risk of consultation (details available in **S2 Fig****).** The effect of each variable on the risk of consultation in occupational health is represented by a dot on a horizontal line in the forest-plot. The dots represent the risk (odds ratio) for each variable, and the length of each line around the dots represent their 95% confidence interval (95 CI). The black solid vertical line represents the null estimate (with a value of 1). Horizontal lines that cross the null vertical line represent non-significant variables on the risk of consultation in occupational health.

Prevalence of workers <30 years old consulting in occupational health was higher during the second and third lockdown (25.9 vs 21.4% and 23.1 vs 20.1% in the control period, respectively, p < 0.05) (S2 Table and Fig 3). Age was associated with a decrease in the propension of consulting for the three lockdown periods (p < 0.001). For each 10-year increase, there was a decrease by 13% for the first lockdown (OR 0.87, 95 CI 0.81 to 0.94), 9% for the second lockdown (0.91, 0.88 to 0.94) and 9% for the third lockdown (0.91, 0.88 to 0.96) (Fig 4 and S2 Fig). It should be noted that for the first lockdown, this propension was more pronounced for consultations made by nurses, that decreased by 32% (0.68, 0.51 to 0.91, p = 0.009) (S3 and S4 Figs). The prevalence of men was lower during the first lockdown (48.7 vs 57.6% in the control period, p < 0.001) (S2 Table and Fig 3). The risk of consulting for men versus women was significant only for the first lockdown with a decrease of 19% (0.81, 0.68 to 0.98, p = 0.026) (Fig 4 and S2 Fig). The prevalence of consultations for workers with a handicap was higher during the first lockdown (8.9 vs 6.5% in the control period, p = 0.022) (S2 Table and Fig 3), but being considered a worker with handicap did not influence the risk of consultation in the multivariate analysis (Fig 4 and S2 Fig).

### Characteristics of consultations

Consultations made by nurses decreased during the three lockdowns. More specifically, only 5% of consultations were made by nurses during the first lockdown (vs 46.7% in the control period, p < 0.001) (S2 Table and Fig 3). The number of consultations by nurses per day per FTE massively dropped by 91% compared to consultations made by physicians during the first lockdown (OR 0.09, 95 CI 0.06 to 0.13, p < 0.001). Then, the number of consultations made by nurses was still decreased compared to those made by physicians but in lower proportion (0.81, 0.74 to 0.89 during the second lockdown, and 0.50, 0.44 to 0.57 during the third lockdown; p < 0.001) (Fig 4 and S2 Fig). The prevalence of consultations for workers working in essential sectors was higher during the first lockdown (52.5 vs 38.5% in the control period, p < 0.001) (S2 Table and Fig 3). Workers belonging to essential sectors of activity were more prone to have a consultation during the first (1.58, 1.32 to 1.89, p < 0.001) and third lockdown (1.20, 1.07 to 1.34, p = 0.002). The same trends were observed for consultations by physicians, while for nurses no significant variation was observed (S3 and S4 Figs). By law, return-to-work consultations are only made by physicians. The prevalence of return-to-work consultations was massively higher during the first lockdown (61.1 vs 22.9% in the control period, p < 0.001) (S2 Table and Fig 3). The proportion of consultations for return-to-work was multiplied by 2.44 (2.02 to 2.95, p < 0.001) during the first lockdown whereas it decreased by 16% during the second lockdown (0.84, 0.74 to 0.95, p = 0.006) and by 38% during the third lockdown (0.62, 0.53 to 0.73, p < 0.001) (Fig 4 and S2 Fig).

### Conclusions of consultations

No significant difference was found for consultations with workstation layout. The prevalence of return-to-work consultations tended to be lower was during the first lockdown (2.3 vs 3.7%, p = 0.084) and was lower during the second lockdown (2.7% vs 4.8% in the control period, p < 0.001) (S2 Table and Fig 3). A reduced risk of being declared unfit to work was marked during the first (OR = 0.48, 95 CI 0.27 to 0.84, p = 0.010) and second lockdown (0.61, 0.45 to 0.84, p = 0.002) (Fig 4 and S2 Fig). The prevalence of consultations with orientation was lower during the first lockdown (5.4 vs 9.0% in the control period, p = 0.002) (S2 Table and Fig 3). A reduced risk of having orientation following the consultation was observed during the first lockdown (0.53, 0.37 to 0.77, p = 0.001) (Fig 4 and S2 Fig). The nurse consultations were marked by a decrease in consultations with orientation during the three lockdowns. There was no orientation made by nurses during the first lockdown (OR not computable), and the consultations with orientation decreased by 29% (0.71, 0.54 to 0.94, p = 0.018) during the second lockdown and by 35% (0.65, 0.45 to 0.94, p = 0.023) during the third lockdown (S4 Fig).

## Discussion

The main findings were that the Covid-19 pandemic had a huge impact on the medical activity of occupational health departments, with a massive decrease in the number of consultations in 2020 followed by an increase in 2021 compared to 2019. Workers from essential sectors, youngers, and women, had a higher propensity to consult during the first lockdown. Return-to-work consultations increased during the first lockdown, but the risk of being unfit to work was lower.

### The massive impact of the pandemic on occupational health consultations

Our study showed a 15% decrease in occupational health consultations in 2020 compared to 2019. To our knowledge, only one study in Poland evaluated the negative impact of the pandemic on access to and utilization of occupational health services and showed a 25% decrease in occupational health consultations [10]. This difference of impact of Covid-19 on occupational health activity may be linked with heterogeneous impact of Covid between countries, that was particularly evident at the beginning of the pandemic [21]. The study in Poland only compared mean annual activity between 2019 and 2020 [10], whereas our study analyzed three consecutive years, with longitudinal time-series analysis, acute effects of lockdowns, and putative influencing variables. In particular, we showed an eight-time drop during the first lockdown. This drop may be due to a variety of factors, such as the closure of workplaces [22], restrictions on movement [3], prioritization of Covid-related cases, and fear of contracting Covid-19 [23]. Teleconsultation may appear as a solution, facilitating ongoing care and accessibility to occupational health services despite movement restrictions. However, although some occupational departments had the technology for teleconsulting, not all consultations could be carried out virtually, and some patients preferred in-person visits [24]. We also showed a rebound in occupational consultations in 2021 compared to 2019. To our knowledge, there are no other studies that examined this rebound in occupational health activity. However, similar effects were observed in other healthcare settings and sectors of activity [10]. For occupational health consultations, the increase observed in 2021 may be due to several factors. Covid-19 vaccines contributed to normalize working conditions [25], as well as the lifting of lockdown restrictions [26]. Lastly, occupational departments also wanted to catch-up the consultations that were not be carried out in 2020 [27].

### Characteristics of consultations and influencing variables

Age was negatively associated with the propensity of consulting for all three lockdown periods. Considering that older individuals being at higher risk for Covid-19, they may have been more hesitant to seek medical attention [28]. Interestingly, middle-aged workers were those who reported high sick leave, with also greater incidence of contact sick leaves [29]. The finding that more women sought occupational health services during the first lockdown compared to men could be related to differences in job types, work arrangements, or caregiving responsibilities [30]. In particular, essential activity sectors during the Covid pandemic were medico-social sector and large-scale distribution, that are composed of a majority of female workers [31]. In our study, a handicap had no impact on consultations during the three lockdowns, contrary to other studies that demonstrated a decline in occupational interventions for people with disabilities [32] and an increase in prescriptions [33]. A meta-analysis also demonstrated the barriers faced by people with disabilities during the Covid-19 pandemic [34]. The finding that there were more return to work visits during the first lockdown may be linked with more illnesses, requiring a medical clearance for return to work [35,36]. Return to work visits were also proportionally predominant during the first lockdown because at the beginning of the pandemic only consultations requiring medical supervision were maintained. Paradoxically, we showed a low number of unfitness to work during the first lockdown which is counterintuitive, as return visits are typically associated with higher rates of unfitness to work [37]. In the first lockdown, there was also a decrease in consultations without orientation, that may be linked with access restrictions to non-essential healthcare [38]. However, subsequent lockdowns showed progress with the introduction and expansion of telehealth services [26], that may explain the normalization of consultations without orientation.

### Limitations

Our study has some limitations. Considering the massive set of data (112,082 consultations), we did not include all text field nor uncertain data. Information about health issues and working conditions that have provoked the consultation were also a text field, and thus not included. The retrospective design might be seen as a limitation because it relies on looking back at past data, that are traditionally more subjected to bias. Even if the population and working conditions may differ from other regions and preclude generalizability [39]. we included more than 100 000 consultations with all socio-professional categories. The retrospective design did not prove causality. Further studies should assess the risk factors for being unfit to work during the pandemic, with a focus on modifications of occupational exposures.

## Conclusion

In conclusion, the Covid-19 pandemic significantly affected the occupational health departments, causing a substantial decrease in consultations in 2020, followed by an increase in 2021 compared to 2019. Essential sector workers, younger individuals, and women had a higher propensity to consult during the initial lockdown. Return-to-work consultations increased during the first lockdown, with a lower risk of being unfit to work. The findings underscored the pandemic’s profound impact on occupational health and call for further research to understand its implications.

## Supporting information

S1 Table Average number of consultations per day and per full-time equivalent occupational healthcare worker by year, and comparisons between years (Hedge’s bias corrected effect size and its 95% confident intervals; effect-sizes are considered small between 0.20 and 0.50, moderate between 0.50 and 0.80, and high above 0.80). (TIF)

S2 Table Number of consultations (%) during each lockdown period compared to a control period (one year before for the first and second lockdown, and two years before for the third lockdown). *Comparisons were made using chi-square test or Fisher exact test when appropriate:* ^†^ *p < 0.10*, * p < .05, ** p < .01, *** p < .001.
(TIF)

S1 Fig Evolution of the number of consultations per day and per full-time equivalent occupational physician and by full-time equivalent occupational health nurse. (TIF)

S2 Fig Variables influencing the risk of consultation at the occupational health department. (TIF)

S3 Fig Variables influencing the risk of consultation by an occupational physician. N/A: not applicable. (TIF)

S4 Fig Variables influencing the risk of consultation by an occupational health nurse. (TIF)

S1 Supporting information Anonymized dataset underlying the study’s findings. (CSV)

## References

[1] Radević I, Alfirević N, Lojpur A. Corruption, public trust and medical autonomy in the public health sector of Montenegro: taking stock of the COVID-19 influence. PLoS One. 2022;17(9):e0274318. doi: 10.1371/journal.pone.0274318 36074759 PMC9455845

[2] Bouillon-Minois J-B, Schmidt J, Dutheil F. SARS-CoV-2 pandemic and emergency medicine: the worst is yet to come. Am J Emerg Med. 2021;42:246–7. doi: 10.1016/j.ajem.2020.06.014 32593460 PMC7286255

[3] Chang H, Kang MW, Paek SH. Impact of the COVID-19 pandemic on emergency department utilization patterns in South Korea: a retrospective observational study. Medicine (Baltimore). 2022;101(8):e29009. doi: 10.1097/MD.0000000000029009 35212313 PMC8878873

[4] Inama M, Casaril A, Alberti L, Cappellari TF, Impellizzeri HG, Bacchion M, et al. Reduction of the emergency activity, during the Covid 19 Italian lockdown, what’s the lesson to learn?. Health Policy. 2021;125(9):1173–8. doi: 10.1016/j.healthpol.2021.07.013 34373110 PMC8327612

[5] Lee DJ, Shelton JB, Brendel P, Doraiswami R, Makarov D, Meeks W, et al. Impact of the COVID-19 pandemic on urological care delivery in the United States. J Urol. 2021;206(6):1469–79. doi: 10.1097/JU.0000000000002145 34470508 PMC8584199

[6] Patel VM, Kominsky E, Tham T, Bottalico D, Setzen M, Ferastraoaru D, et al. The impact of the COVID-19 pandemic on otolaryngologic emergency department visits at two major NYC hospital systems. Am J Otolaryngol. 2021;42(5):103123. doi: 10.1016/j.amjoto.2021.103123 34186437 PMC8214322

[7] Maalej R, Hage R, Salviat F, Vignal-Clermont C. Impact of lockdown during the COVID-19 outbreak on ophthalmological emergencies in a referral center in France. J Fr Ophtalmol. 2022;45(1):1–8. doi: 10.1016/j.jfo.2021.10.002 34823891 PMC9304632

[8] Gonçalves-Pinho M, Mota P, Ribeiro J, Macedo S, Freitas A. The impact of COVID-19 pandemic on psychiatric emergency department visits – a descriptive study. Psychiatr Q. 2021;92(2):621–31. doi: 10.1007/s11126-020-09837-z 32839923 PMC7445073

[9] de Oliveira M, Fuller T, Gabaglia C, Cambou M, Brasil P, de Vasconcelos Z. Repercussions of the COVID-19 pandemic on preventive health services in Brazil. Prev Med. 2022;155:106914. 34953811 10.1016/j.ypmed.2021.106914 PMC8716082

[10] Marcinkiewicz A. The impact of the COVID-19 pandemic on the resources and activities of basic occupational health services in Poland. Med Pr. 2022;73(1):19–24. doi: 10.13075/mp.5893.01217 35068486

[11] Alessio L, Imbriani M. Report on the activity of the Lombardy Association of Occupational Medicine and Industrial Hygiene (ALMLII) in 1998–2000. Ital Med Lav Ergon 2001;23(1):71–4. 11386192

[12] Institute for Medical Research and Occupational Health. Institute for Medical Research and Occupational Health, activity report for 2006. Arh Hig Rada Toksikol. Mars 2007;58(1):89–171. 17424788

[13] Cox RAF, Edwards F, Palmer K, Royal College of Physicians of London Faculty of Occupational Medicine. In: Cox RAF, Edwards FC, Palmer K, eds. Fitness for Work: The Medical Aspects, 3rd ed. Oxford, New York: Oxford University Press; 2000. xxii+618 (Oxford Medical Publications).

[14] Fondow M, Zeidler Schreiter E, Thomas C, Grosshans A, Serrano N, Kushner K. Initial examination of characteristics of patients who are high utilizers of an established primary care behavioral health consultation service. Fam Syst Health. 2017;35(2):184–92. doi: 10.1037/fsh0000266 28617019

[15] Turchi V, Verzuri A, Nante N, Napolitani M, Bugnoli G, Severi F. Night work and quality of life. A study on the health of nurses. Ann Ist Super Sanita. 2019;55(2):161–9. 31264639 10.4415/ANN_19_02_08

[16] Tamin J. COVID-19 and vulnerable workers. Occup Med. 2021;71(3):161. 10.1093/occmed/kqab016 PMC7928656 33585919

[17] Romero DE, Muzy J, Damacena GN, de Souza NA, de Almeida WS, Szwarcwald CL. Older adults in the context of the COVID-19 pandemic in Brazil: effects on health, income and work. Cad Saude Publica. 2021;37(3):e00216620. 10.1590/0102-311X00216620 33825801

[18] Farajallah HM, AlSuwaidi SK, AlSuwaidi SM, AlAli GA, AlZubaidi AS, Carrick FR, et al. Large variations in disease severity, death and ICU admission of 2993 patients infected with SARS-CoV-2: The potential impact of genetic vulnerability. J Infect Public Health. 2021;14(7):886–91. doi: 10.1016/j.jiph.2021.04.008 34118740 PMC8061633

[19] Porto ED, Naticchioni P, Scrutinio V. Lockdown, essential sectors, and Covid-19: lessons from Italy. J Health Econ. 2022;81:102572. 34958981 10.1016/j.jhealeco.2021.102572 PMC8648381

[20] Citrome L, Magnusson K. Paging C. An effect size interpretation is required STAT!: visualising effect size and an interview with Kristoffer Magnusson. Int J Clin Pract. 2014;68(5):533–4. doi: 10.1111/ijcp.12435 24750523

[21] Kontopantelis E, Mamas MA, Webb RT, Castro A, Rutter MK, Gale CP, et al. Excess deaths from COVID-19 and other causes by region, neighbourhood deprivation level and place of death during the first 30 weeks of the pandemic in England and Wales: a retrospective registry study. Lancet Reg Health Eur. 2021;7:100144. doi: 10.1016/j.lanepe.2021.100144 34557845 PMC8454637

[22] Birimoglu Okuyan C, Begen MA. Working from home during the COVID-19 pandemic, its effects on health, and recommendations: the pandemic and beyond. Perspect Psychiatr Care. 2022;58(1):173–9. doi: 10.1111/ppc.12847 34003489 PMC8242705

[23] Dubey S, Biswas P, Ghosh R, Chatterjee S, Dubey MJ, Chatterjee S, et al. Psychosocial impact of COVID-19. Diabetes Metabol Syndr: Clin Res Rev. 2020;14(5):779–88. 10.1016/j.dsx.2020.05.035 PMC7255207 32526627

[24] Gupta N, Balcom SA, Gulliver A, Witherspoon RL. Health workforce surge capacity during the COVID-19 pandemic and other global respiratory disease outbreaks: a systematic review of health system requirements and responses. Health Plann Manage. 2021;36(S1):26–41. doi: 10.1002/hpm.3137

[25] Giannitrapani KF, Brown-Johnson C, Connell NB, Yano EM, Singer SJ, Giannitrapani SN, et al. Promising strategies to support COVID-19 vaccination of healthcare personnel: qualitative insights from the VHA national implementation. J Gen Intern Med. 2022;37(7):1737–47. doi: 10.1007/s11606-022-07439-y 35260957 PMC8902903

[26] Garzillo EM, Cioffi A, Carta A, Monaco MGL. Returning to work after the COVID-19 pandemic earthquake: a systematic review. Int J Environ Res Public Health. 2022;19(8):4538. doi: 10.3390/ijerph19084538 35457407 PMC9024882

[27] Barro K, Malone A, Mokede A, Chevance C. Management of the COVID-19 epidemic by public health establishments-Analysis by the Fédération Hospitalière de France. J Visc Surg. 2020;157(3S1):S19–23. doi: 10.1016/j.jviscsurg.2020.04.011 32417194 PMC7166021

[28] Hu B, Guo H, Zhou P, Shi Z-L. Characteristics of SARS-CoV-2 and COVID-19. Nat Rev Microbiol. 2021;19(3):141–54. doi: 10.1038/s41579-020-00459-7 33024307 PMC7537588

[29] Smith DRM, Jijón S, Oodally A, Shirreff G, Aït Bouziad K, Ante-Testard PA, et al. Sick leave due to COVID-19 during the first pandemic wave in France, 2020. Occup Environ Med. 2023;80(5):268–72. doi: 10.1136/oemed-2022-108451 36914254 PMC10176331

[30] Gaitens J, Condon M, Fernandes E, McDiarmid M. COVID-19 and essential workers: a narrative review of health outcomes and moral injury. Int J Environ Res Public Health. 2021;18(4):1446. doi: 10.3390/ijerph18041446 33557075 PMC7913818

[31] Apple R, O’Brien EC, Daraiseh NM, Xu H, Rothman RL, Linzer M, et al. Gender and intention to leave healthcare during the COVID-19 pandemic among U.S. healthcare workers: a cross sectional analysis of the HERO registry. PLoS One. 2023;18(6):e0287428. doi: 10.1371/journal.pone.0287428 37327216 PMC10275433

[32] Sánchez-Guarnido AJ, Domínguez-Macías E, Garrido-Cervera JA, González-Casares R, Marí-Boned S, Represa-Martínez Á, et al. Occupational therapy in mental health via telehealth during the COVID-19 pandemic. Int J Environ Res Public Health. 2021;18(13):7138. doi: 10.3390/ijerph18137138 34281072 PMC8297153

[33] Rauf B, Sheikh H, Majid H, Roy A, Pathania R. COVID-19-related prescribing challenge in intellectual disability. BJPsych Open. 2021;7(2):e66. doi: 10.1192/bjo.2021.26 33736746 PMC8058854

[34] Croft S, Fraser S. A scoping review of barriers and facilitators affecting the lives of people with disabilities during COVID-19. Front Rehabil Sci. 2022;2:784450. doi: 10.3389/fresc.2021.784450 36188856 PMC9397712

[35] Marconi AM, Myers U, Retamar AM, Freddi IJ, Chiarelli J, Zamora R. Increase in use of psychiatric sick leave during COVID-19 pandemic by healthcare workers in a municipality in Argentina. Rev Bras Med Trab. 2022;20(1):36–44. 36118070 10.47626/1679-4435-2022-872 PMC9444219

[36] Palladino R, Mercogliano M, Fiorilla C, Frangiosa A, Iodice S, Sanduzzi Zamparelli S, et al. Association between COVID-19 and sick leave for healthcare workers in a large academic hospital in Southern Italy: an observational study. Int J Environ Res Public Health. 2022;19(15):9670. doi: 10.3390/ijerph19159670 35955042 PMC9368056

[37] McEwan IM. Absenteeism and sickness absence. Postgrad Med J. 1991;67(794):1067–71. 1800967 10.1136/pgmj.67.794.1067 PMC2399182

[38] Moynihan R, Sanders S, Michaleff ZA, Scott AM, Clark J, To EJ, et al. Impact of COVID-19 pandemic on utilisation of healthcare services: a systematic review. BMJ Open. 2021;11(3):e045343. doi: 10.1136/bmjopen-2020-045343 33727273 PMC7969768

[39] McGue M, Osler M, Christensen K. Causal inference and observational research: the utility of twins. Perspect Psychol Sci. 2010;5(5):546–56. doi: 10.1177/1745691610383511 21593989 PMC3094752

