# Supplementary figures and images for: Impact of COVID on the medical activity of occupational health departments
Source: PLoS One. 2025 May 22;20(5):e0323018. doi: 10.1371/journal.pone.0323018 (PMC12097605; doi:10.1371/journal.pone.0323018)

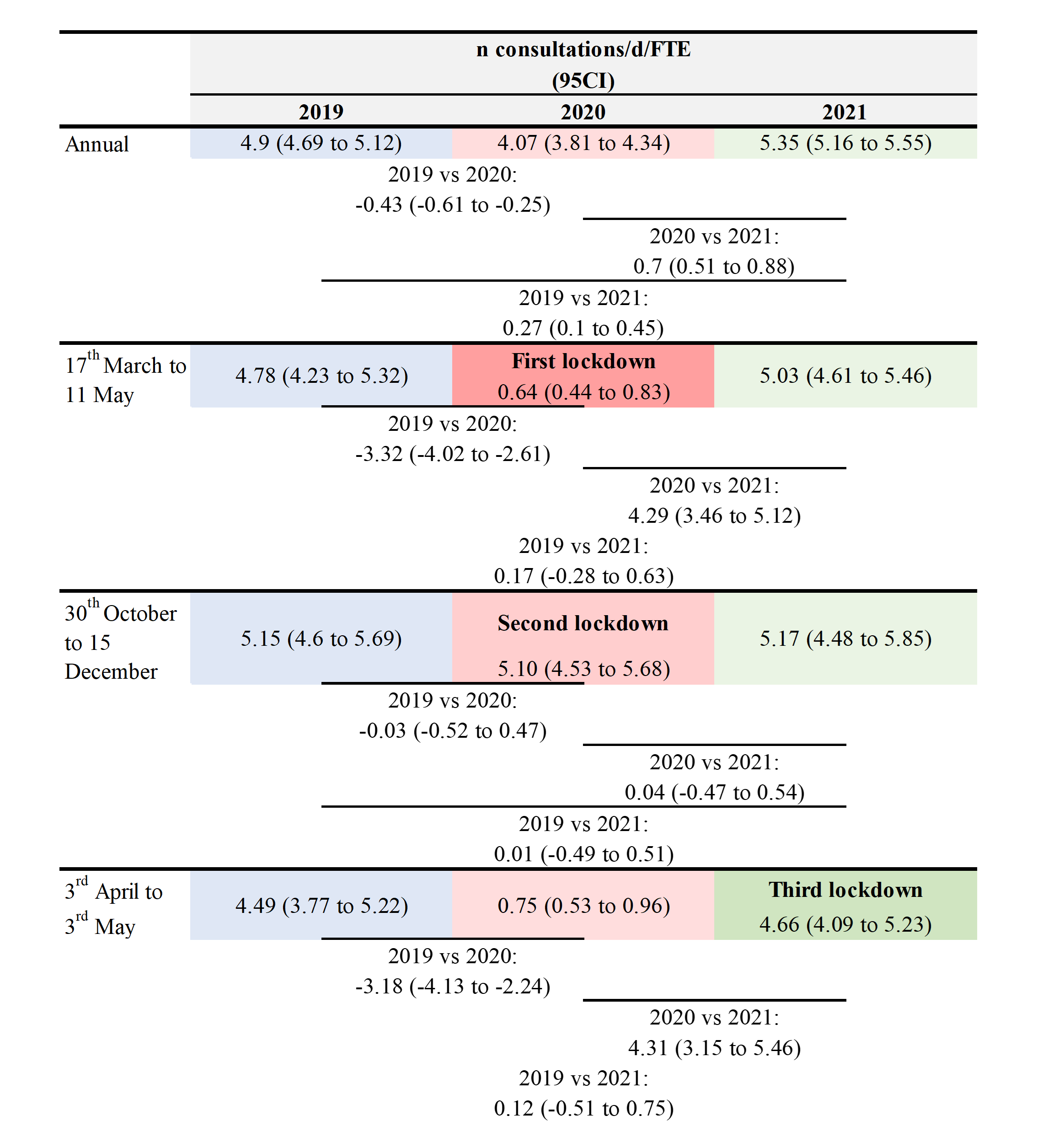

Supplement: S1 Table — (TIF) [file pone.0323018.s001.tif]

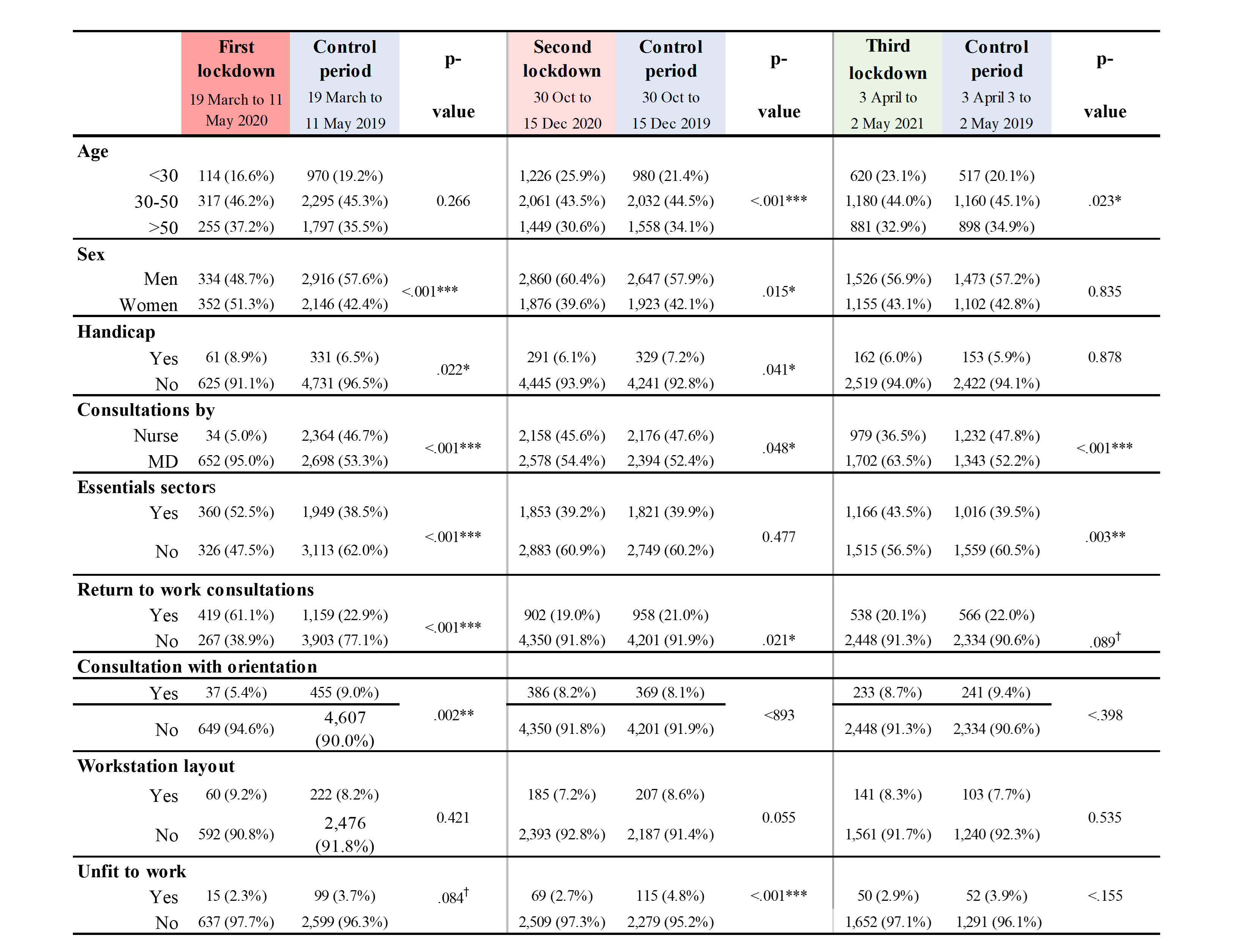

Supplement: S2 Table — Comparisons were made using chi-square test or Fisher exact test when appropriate: † p < 0.10, * p < .05, ** p < .01, *** p < .001. (TIF) [file pone.0323018.s002.tif]

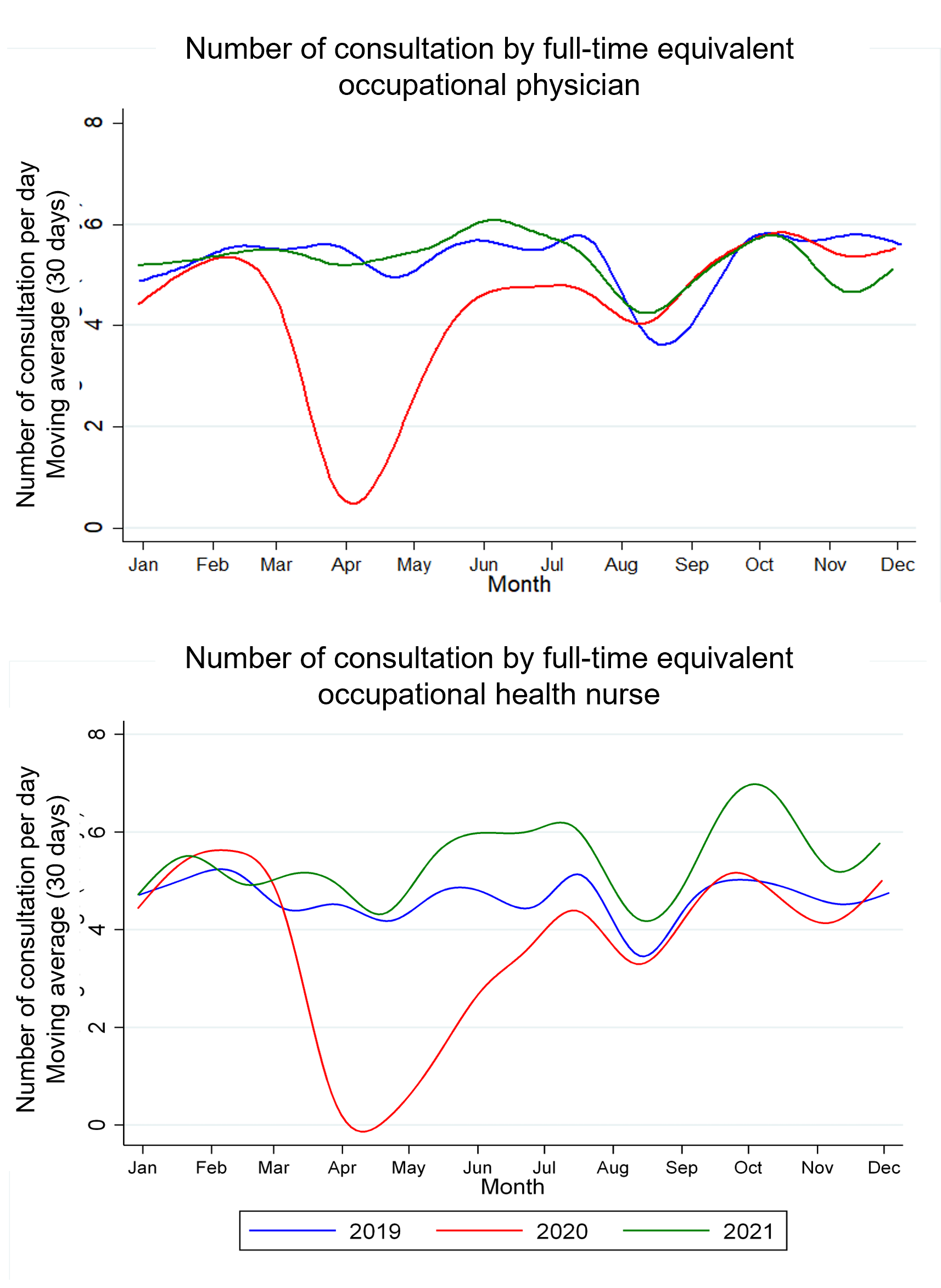

Supplement: S1 Fig — (TIF) [file pone.0323018.s003.tif]

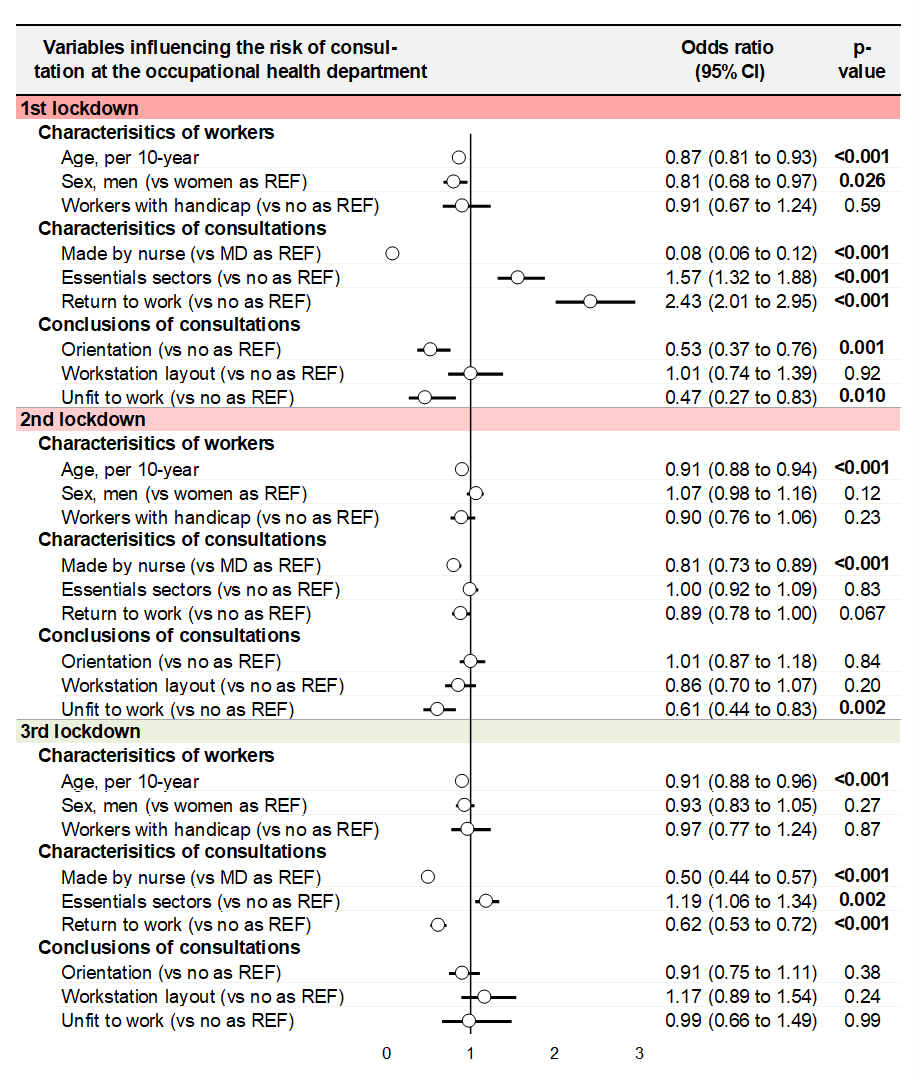

Supplement: S2 Fig — (TIF) [file pone.0323018.s004.tif]

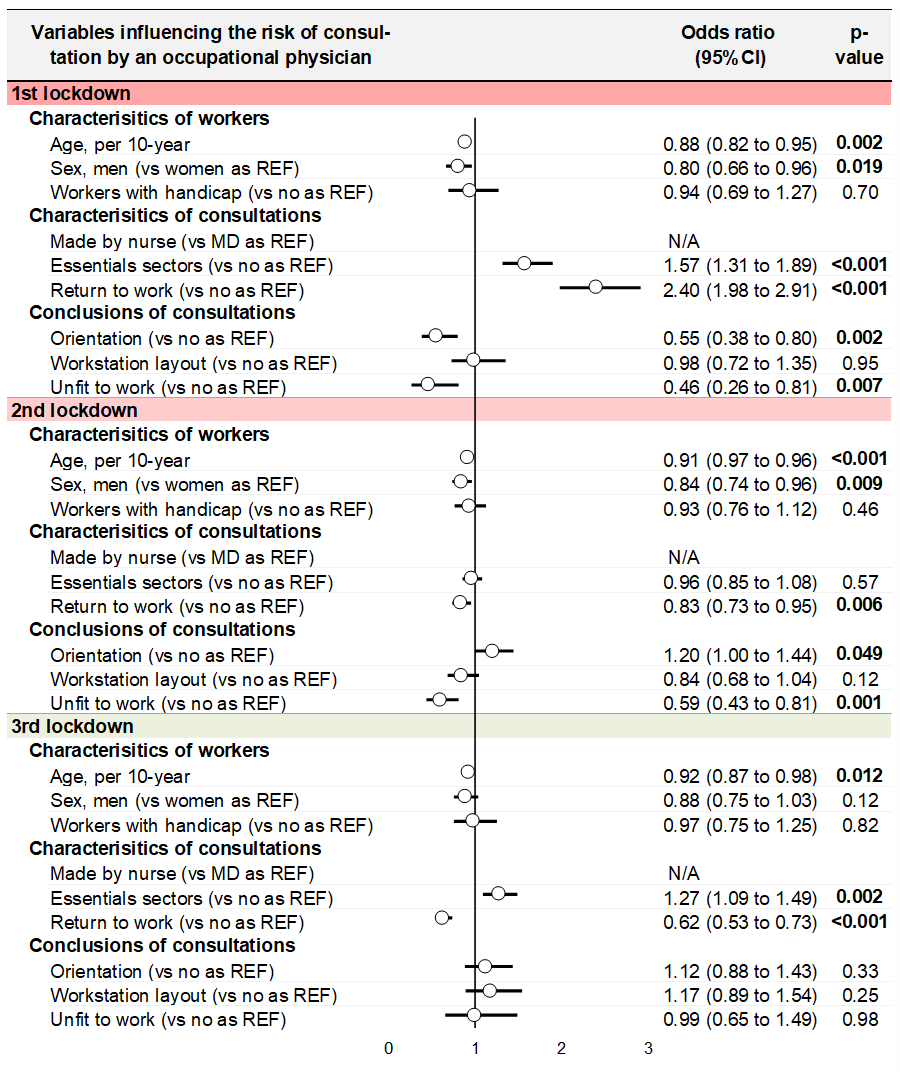

Supplement: S3 Fig — (TIF) [file pone.0323018.s005.tif]

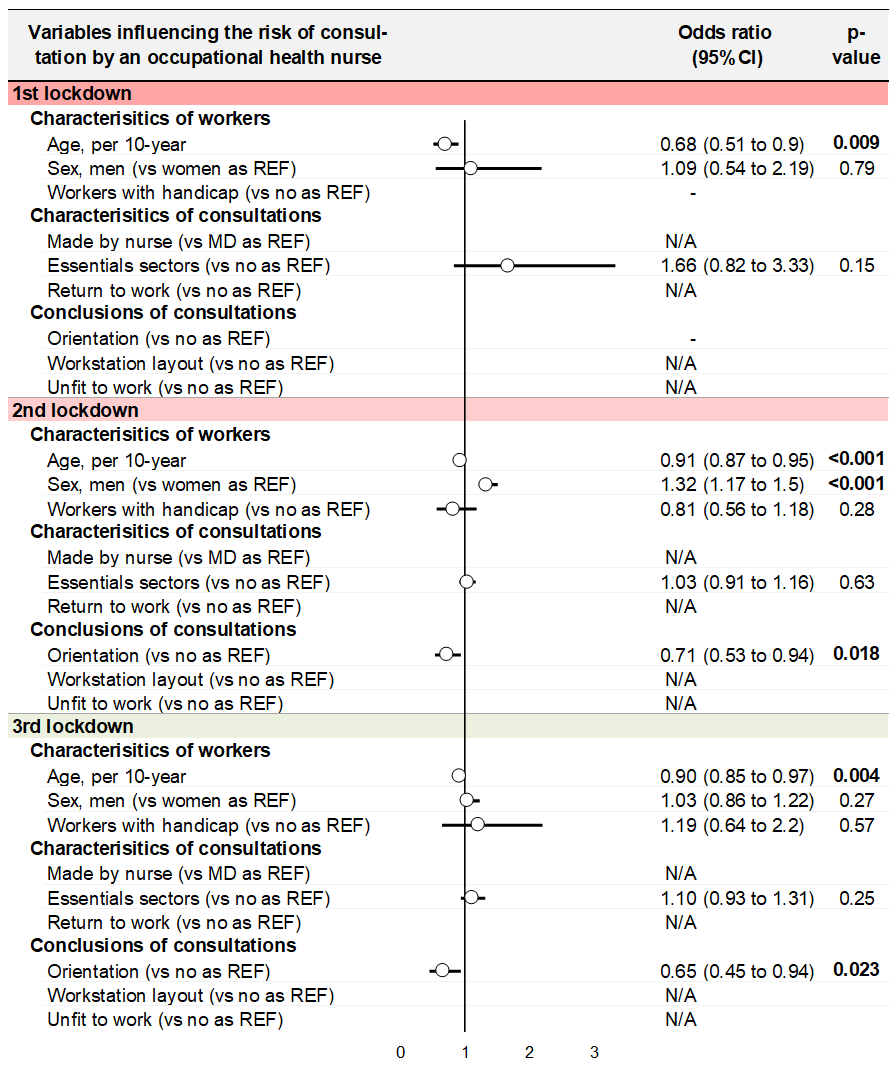

Supplement: S4 Fig — (TIF) [file pone.0323018.s006.tif]
